# Regulation of ACE-2 enzyme by hyperoxia in lung epithelial cells by post-translational modification

**Published:** 2021-05-06

**Authors:** Tarek Mohamed, Amal Abdul-Hafez, Bruce D Uhal

**Affiliations:** 1 Department of Pediatrics and Human Development, Michigan State University, USA; 2 Department of Physiology, Michigan State University, USA

**Keywords:** angiotensin converting enzyme-2 (ACE-2), renin angiotensin system, bronchopulmonary dysplasia, ubiquitin proteasome system, hyperoxia

## Abstract

**Background:**

Bronchopulmonary Dysplasia (BPD) occurs in premature neonates with respiratory distress who require supplemental oxygen in the first days after birth. BPD involves uniform arrest of alveolar development and variable interstitial cellularity and/or fibroproliferation. Previous studies by our lab showed that the enzyme, angiotensin converting enzyme-2 (ACE-2) and its product Ang1-7 exerting action on the receptor Mas oncogene in what is known as ACE-2/Mas axis is protective to lung cells. We also showed that ACE-2 is expressed in fetal human lung fibroblasts but is significantly decreased by hyperoxic gas lung injury, an effect caused by ACE-2 enzyme shedding mediated by TNF-alpha-converting enzyme (TACE/ADAM17). However, no reports yet exist about the regulation of ACE-2 in the alveolar epithelia in hyperoxic lung injury.

**Objective:**

In this study we aim to define the effects of hyperoxic lung injury on the protective ACE-2 enzyme in the human lung alveolar epithelial cell line A549.

**Design/Methods:**

Cultured A549 cells were exposed to hyperoxia (95% O2) or normoxia (21% O2) for 3 or 7 days in serum-free nutrient media. Cells were lysed and culture media were collected to test for cellular ACE-2 enzymatic activity and for ACE-2, Mas receptor, TACE/ADAM17, and ubiquitin proteins abundance by immunoblotting. Cells were harvested in Trizol for RNA extraction and ACE-2 qRT-PCR. Whole cell extracts of A549 cell line was used for ACE-2 immunoprecipitation and subsequent ubiquitin immunoblotting.

**Results:**

Total ubiquitinated proteins were increased by hyperoxia treatment, while ACE-2 and Mas receptor proteins abundance and ACE-2 enzymatic activity were decreased significantly in A549 cells exposed to hyperoxia relative to the normoxia controls. The percent decrease in ACE-2 activity corresponded with increased time of hyperoxic gas exposure. However, in contrast to our data from lung fibroblasts, no significant change was noted in ACE-2 protein released into the media or in ACE-2 mRNA levels by the hyperoxic treatment. Ubiquitin immunoreactive bands were detectable in the ACE-2 immunoprecipitate.

**Conclusion(s):**

These data suggest that hyperoxic exposure of the lung epithelial cells decreases the protective enzyme ACE-2 by cell type specific mechanisms independent of shedding by TACE/ADAM17. The data also suggest a regulatory level of ACE-2 downstream of transcription may involve ACE-2 ubiquitination and targeting for degradation.

## Introduction

Bronchopulmonary Dysplasia (BPD) is a problem that faces premature neonates who present with respiratory distress and require supplemental oxygen and mechanical ventilation in the first days after birth, causing arrest of alveolar development and variable interstitial cellularity and/or fibroproliferation.^1–4^ BPD is associated with potential multiple complications in the respiratory and nervous system, thus presenting an urgent need to understand mechanisms of BPD.^5^

Previous studies from our lab and others showed that angiotensin converting enzyme-2 (ACE-2) is protective to lung cells in multiple disease conditions including pulmonary fibrosis and BPD.^1,6–9^ ACE-2 has been recently the focus in research as it acts as the receptor of SARS-CoV-2 virus causing the recent COVID-19 pandemic. The importance of ACE-2 does not only lie in its role as a viral entry receptor, but also as a lung protective enzyme leading to a further complicated role in lung disease.^10^ The enzyme ACE-2 produces the peptide product Ang1-7 that exerts its action on the receptor Mas oncogene in what is known as ACE-2/Ang1-7/Mas axis. This ACE-2/Ang1-7/Mas axis has been shown to be protective to lung cells.^11,12^ We previously showed that ACE-2 is expressed in fetal human lung fibroblasts and that hyperoxia significantly decreases ACE-2 enzyme levels. This decrease is attributed to enzyme shedding mediated by TNF-alpha-converting enzyme (TACE/ADAM17).^8^ However, prior hypoxia exposure eliminated this hyperoxia-induced decrease in ACE-2 in fetal lung fibroblasts.^2^ In models of BPD, hyperoxia has been suggested to increase ubiquitination and proteasome degradation of some proteins.^13,14^ ACE-2 ubiquitination has been shown to be involved in pulmonary hypertension and neurogenic hypertension.^15,16^

Given the important roles of ACE-2 in protecting against lung injury and of the alveolar epithelial cells in BPD pathogenesis, it becomes important to understand the effects of hyperoxia on ACE-2 enzyme in alveolar epithelial cells as we present in this study.

## Materials and Methods

### Cell culture

Cultured A549 human lung epithelial cell line was used for controlled oxygen gas exposure in serum-free Ham’s F-12 media. The hyperoxia cell group was incubated under flow of 95% O_2_ and 5% CO_2_ while the normoxia (control group) was incubated in 21% O_2_ and 5% CO_2_ for the designated time frame (72 hours or 7 days). The media was collected, supplemented with SigmaFast^™^ protease inhibitor cocktail (Millipore Sigma, St. Louis, MO), centrifuged to remove cell debris, and concentrated using Amicon^®^ protein concentration tubes with 10 kDa molecular weight limit (Millipore Sigma). The cells were lysed in an appropriate buffer for the downstream applications.

### Western blotting

Whole cell lysates were prepared in NP-40 protein extraction buffer supplemented with protease inhibitor cocktail. Lysates or concentrated media were separated on 10% SDS-PAGE gels, transferred on PVDF membrane, blocked using 5% NFDM in TBST, and blotted using antibodies for ACE-2 (Abcam, Cambridge, MA), TACE, Ubiquitin, β-actin (Cell Signaling Technologies, Danvers, MA), or Mas (Santa Cruz Biotechnologies, Dallas, Texas). Bands were detected by chemiluminescence using Amersham ECL Prime Western Blotting Detection Reagent (GE Healthcare Life Sciences, Pittsburgh, PA) and autoradiography film exposure. Bands were quantified using ImageJ software.

### ACE-2 Immunoprecipitation

A549 cells were lysed in NP-40 lysis buffer. Cell lysates were used to immunoprecipitated ACE-2 protein using ACE-2 antibody (abcam) and Dynabeads immunoprecipitation kit (Thermo Fisher Scientific, Waltham, MA). The eluted ACE-2 immunoprecipitate was used for ubiquitin western blot as described above.

### ACE-2 Enzyme activity

Protein was extracted from A549 cells in ice-cold ACE-2 assay buffer containing 100 mM Tris-HCl pH 7.4, 1M NaCl, 0.1% Tween-20 as described previously.^9,11^ The enzymatic activity of ACE-2 in the cell lysate was measured immediately after cell lysis by the cleavage of fluorogenic substrate Mca-APK(Dnp) at 40 μM in the presence of 10 μM lisinopril to block ACE activity. Reactions were performed in black microtitre plates at room temperature in a fluorescence microplate reader (FL600 Biotec Fluorescence Reader; BMG, Durham, NC, USA) over 30 min, using excitation and emission wavelengths of 310/320 and 420/450 nm.

### RNA isolation and RT-PCR

Total RNA was extracted from A549 with Trizol Reagent (Invitrogen) and real time RT-PCR was performed as described previously.^9,17^ First-strand cDNA was synthesized from 2 μg of total RNA with Superscript III reverse transcriptase (Thermo Fisher Scientific) and oligo (dT)12–18. Real-time RT-PCR was carried out with cDNA synthesized from 50 ng of total RNA, SYBR Green PCR core reagents (Applied Biosystems, Foster City, CA, USA) according to the manufacturer’s protocol, and 0.2 μM specific primers for human ACE-2 (sense 5’-CATTGGAGCAAGTGTTGGATCTT-3’ and antisense 5’-GAGCTAATGCATGCCATTCTCA-3’) and β-actin (sense 5’-AGGCCAACCGCGAGAAGATGACC-3’ and antisense 5’-GAAGTCCAGGGCGACGTAGC-3’). The PCR thermal profile started with 10 min activation of Taq polymerase at 95°C followed by 40 cycles of denaturation at 94°C for 60 s, annealing at 55°C for 60 s, and extension at 72°C for 60 s, ending with dissociation curve analysis to validate the specificity of the PCR products. Reactions were performed in StepOne Plus Thermal Cycler (Thermo Fisher Scientific) and threshold cycle (Ct) data were collected. The relative ACE-2 expression was normalized to β-actin and calculated with the comparative Ct method of 2^−ΔΔCt^.

## Results

### Hyperoxia reduces cellular ACE-2 protein

**Figure 1:**
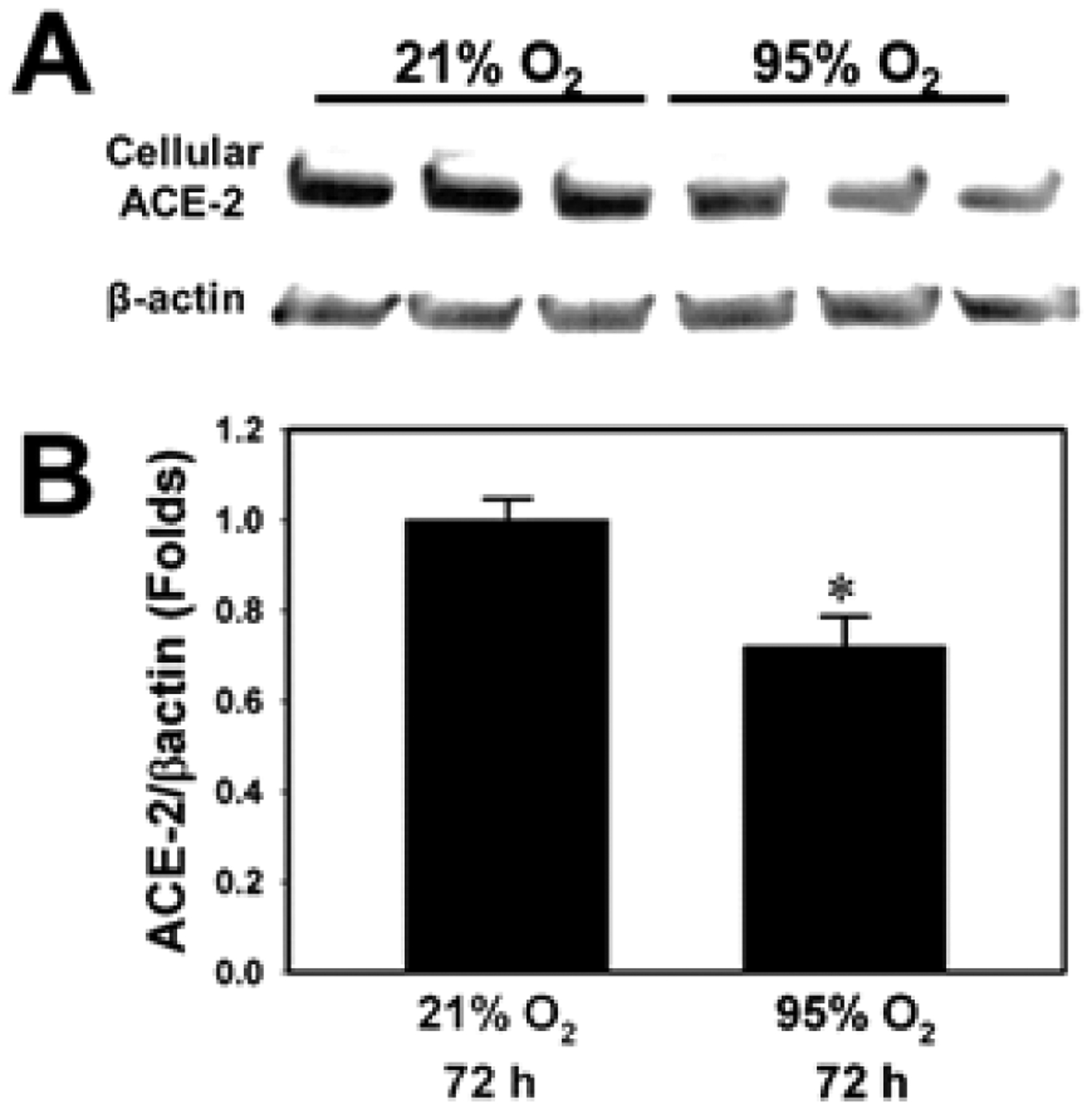
A549 cells were exposed to 95% O_2_ (hyperoxia) or 21% O_2_ (normoxia control). A: Western Blotting for cellular ACE-2 protein normalized to β-actin. B: Densitometry quantification of ACE-2: β-actin western blot bands (*= p<0.05 vs. 21% O_2_ by Student’s t-test, n=3).

Figure 1A shows the results of western blotting for cellular ACE-2 protein normalized to β-actin of A549 cells exposed to 95% O_2_ (hyperoxia) or 21% O_2_ (normoxia control). Densitometry analysis (figure 1B) showed that hyperoxia caused a significant decrease in ACE-2 immunoreactive cellular protein (0.72 fold, p<0.05 vs. 21% O2 by Student’s t-test, n=3).

### Hyperoxia reduces cellular ACE-2 activity

**Figure 2:**
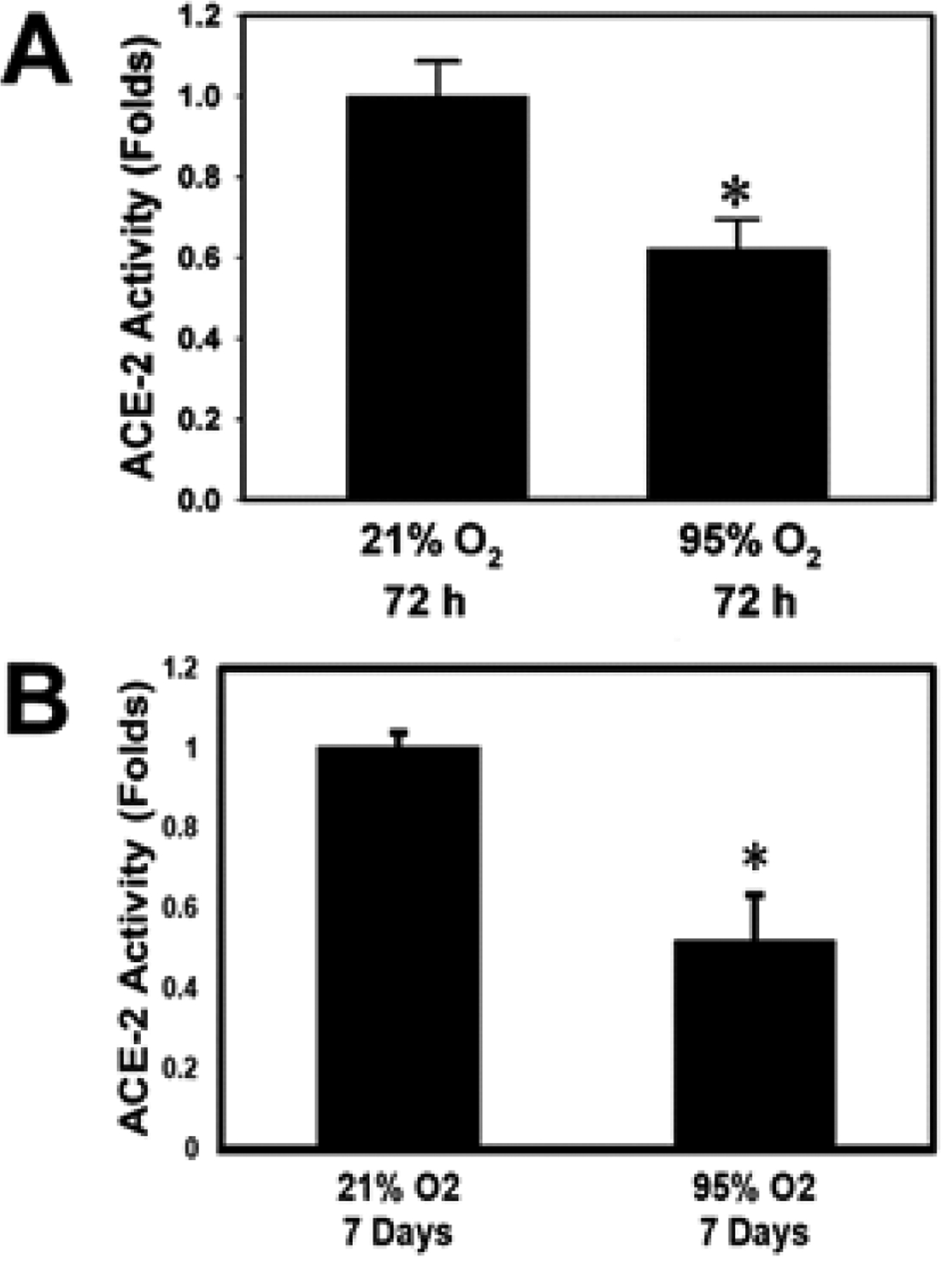
Bar graphs showing ACE-2 enzymatic activity in A549 cells exposed to 95% O_2_ (hyperoxia for A: 72 hours or B: 7 days (*=p<0.05 vs. 21% O_2_ by Student’s t-test, n=3).

In figure 2, ACE-2 enzymatic activity in A549 cell lysates showed a statistically significant reduction (0.62 fold) by 72 hour hyperoxia exposure as well as after 7 days of hyperoxia (0.52 fold) (p<0.05 vs. 21% O2 by Student’s t-test, n=3).

### Hyperoxia does not alter ace-2 mRNA or shed ACE-2 protein

**Figure 3:**
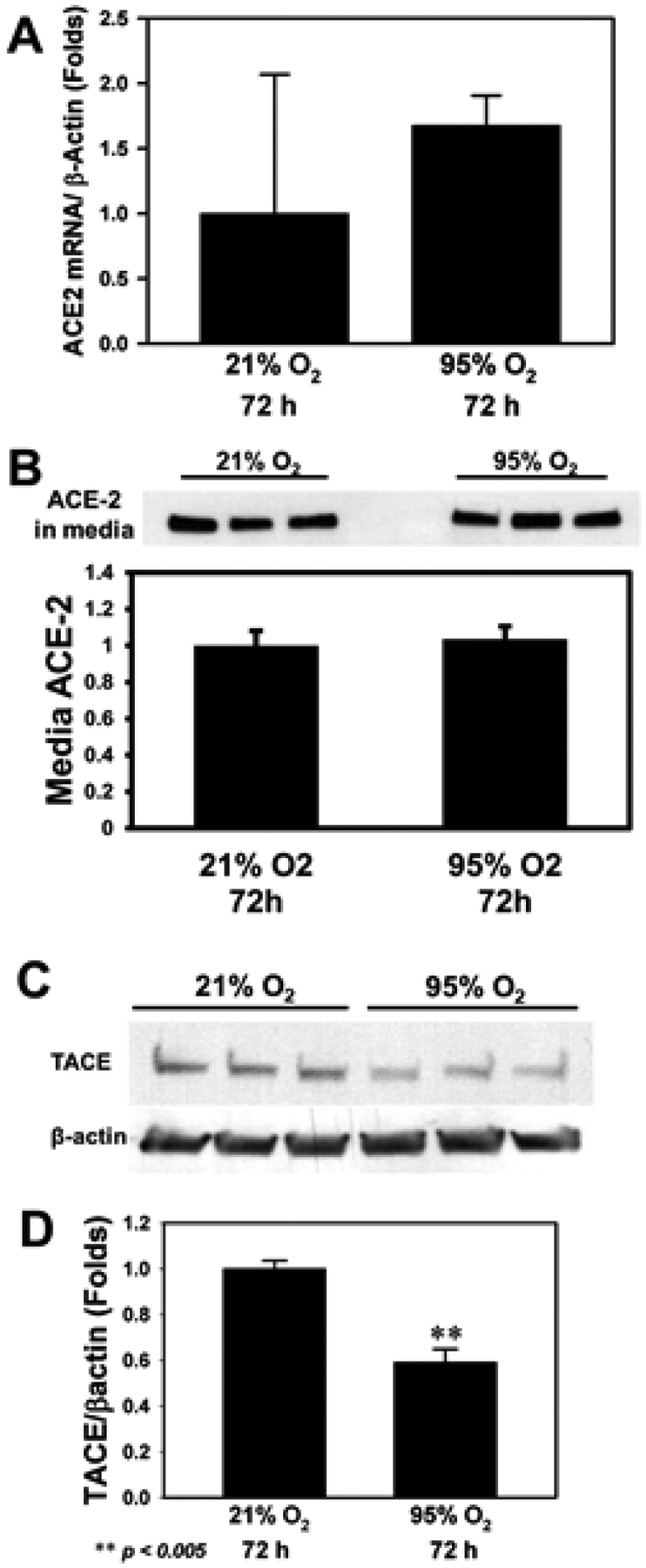
A: ACE-2 mRNA was quantified relative to β-actin using real time RT-PCR in 95% O_2_ (hyperoxia) vs. 21% O_2_ (normoxia) exposed A549 cells. B: Concentrated cell-free culture media supernatants were used for ACE-2 western blotting and analyzed by densitometry (bar graph). C, D: Western Blotting for ADAM17/TACE protein normalized to β-actin (C) and quantification by densitometry (D) (** = p<0.005 vs. 21% O_2_ by Student’s t-test, n=3).

ACE2 mRNA was quantified relative to β-actin using real time RT-PCR (Figure 3A). There was no significant change in relative ACE2 mRNA levels in hyperoxia vs. normoxia exposed cells (n=3). Concentrated cell-free culture media supernatants were used for ACE-2 western blotting (Figure 3B). No significant change is noted in media ACE-2 in hyperoxia treatment compared to the control (n=3). Hyperoxia also reduced the ACE-2 shedding enzyme, ADAM17/TACE in A549. Figure 3C shows western blotting for ADAM17/TACE protein normalized to β-actin. Densitometric analysis in figure 3D shows that hyperoxia caused a significant decrease in ADAM17/TACE (0.6 fold, p <0.005 vs. 21% O2 by Student’s t-test, n=3).

### Evidence of ace-2 ubiquitination

**Figure 4:**
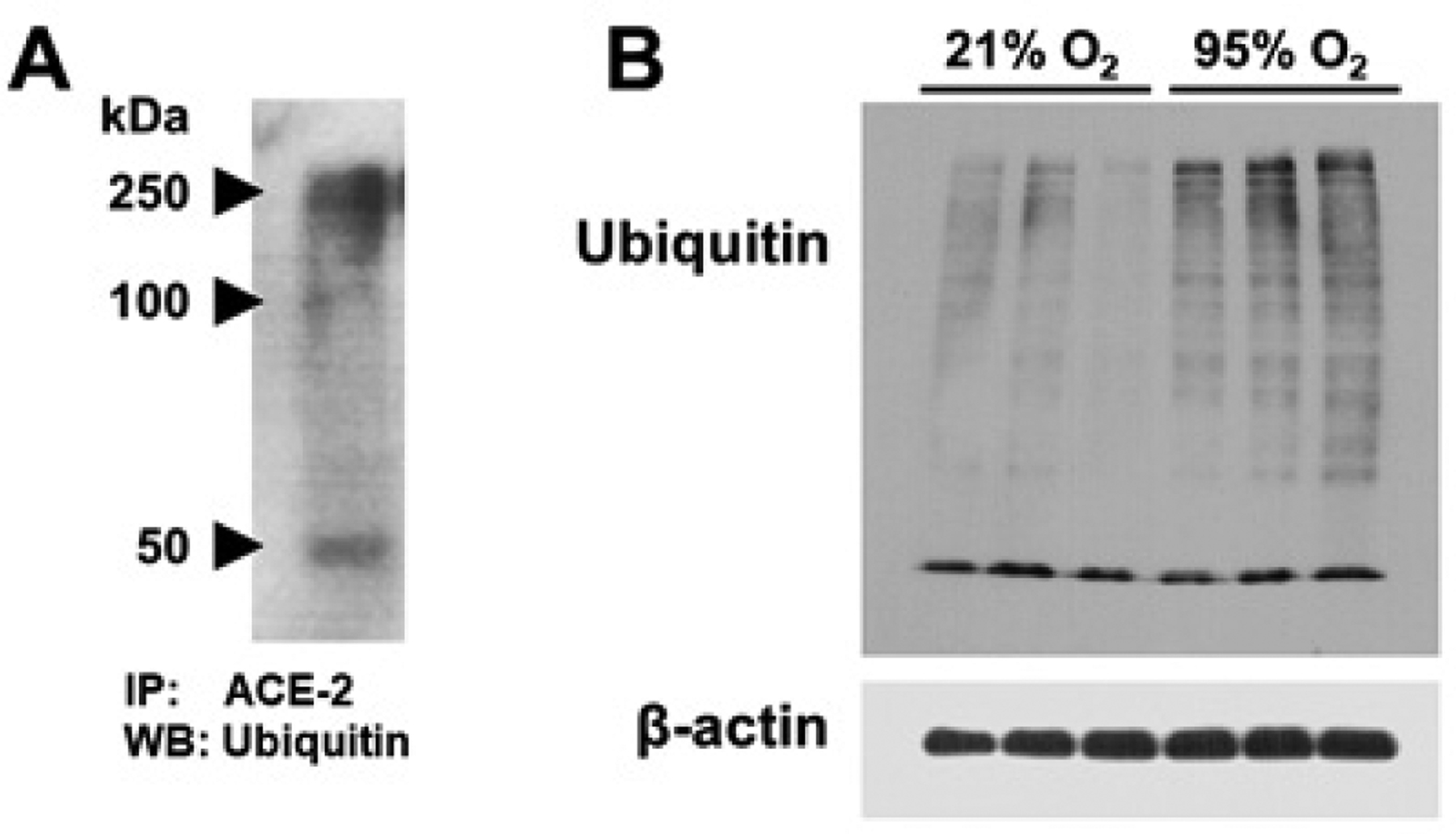
A: A549 cells grown at 21% O_2_ (normoxia) were used for ACE-2 immunoprecipitation. The isolated ACE-2 immuno-precipitate was used for ubiquitin western blotting. B: Western blot for total ubiquitin and β-actin in A549 whole cell lysates of 95% O_2_ (hyperoxia) vs. 21% O_2_ (normoxia) exposed cells.

A549 cell lysate was used for ACE-2 (MW ~100 kDa) immunoprecipitation. The isolated ACE-2 immunoprecipitate was used for ubiquitin western blotting. Figure 4A shows detection of 3 bands at 50, 100 and 250 kDa. This suggests ACE-2 ubiquitination and poly-ubiquitination with subsequent targeting for proteasomal degradation.

### Hyperoxia increases total ubiquitination

Total ubiquitinated proteins in A549 cell lysatess was analyzed by western blotting using anti-ubiquitin antibody. Figure 4B shows that total ubiquitinated proteins in hyperoxia-exposed cells is higher than in normoxia grown cells.

### Hyperoxia increases mas receptor protein

**Figure 5:**
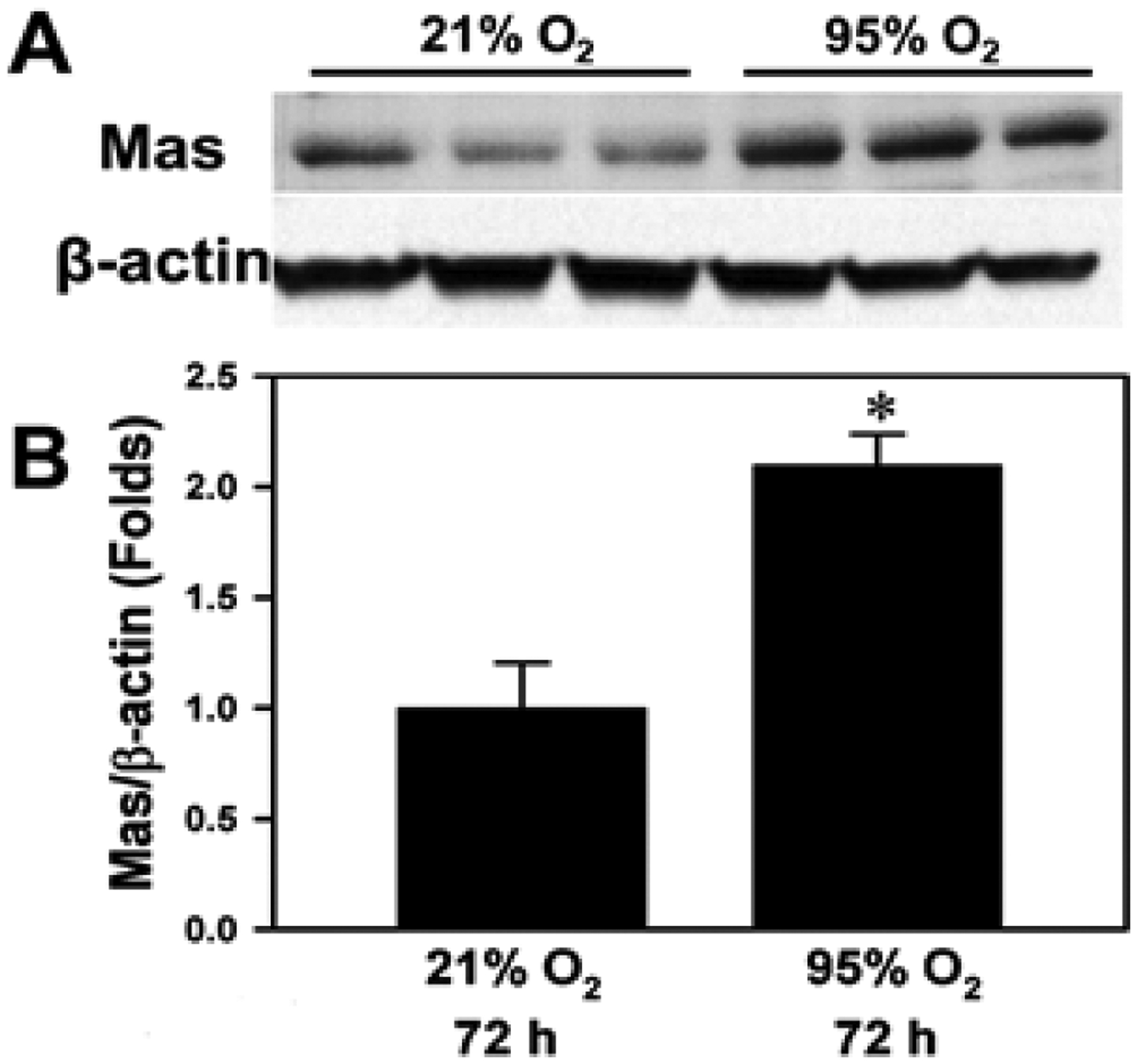
Western Blotting for Mas receptor protein normalized to β-actin (A) and quantification by densitometry (B), 95%O_2_ (hyperoxia) (*=p<0.05 vs. 21% O_2_ by Student’s t-test, n=3).

Figure 5 shows that hyperoxia caused a significant 2-fold increase in Mas receptor protein in A549 cells detected by western blotting and densitometry relative to β-actin (* = p <0.05 vs. 21% O2 by Student’s t-test, n=3).

## Discussion

In our previous study on fetal human lung fibroblasts, we showed that ACE-2 is significantly decreased by hyperoxic gas lung injury due to ACE-2 enzyme shedding mediated by TACE/ADAM17.^8^ Due to the important role of the alveolar epithelia development in hyperoxic lung injury and BPD, we decided to investigate the effects of hyperoxia on ACE-2 in AECs using the human lung alveolar epithelial cell line A549 as a cell culture model. Similar to the lung fibroblasts,^8^ AECs showed reduction in both ACE-2 protein and enzyme activity in response to hyperoxia (Figures 1&2). Thus, we investigated whether ACE-2 mRNA or ACE-2 shedding are the cause of this decrease. Similar to the previous study on fetal fibroblasts, ACE-2 mRNA did not significantly change in response to hyperoxia in AECs (Figure 3A). However, in contrast to the fetal fibroblasts, AECs did not show an effect of hyperoxia on ACE-2 shedding to the culture media (Figure 3B). Furthermore, TACE/ADAM17 protein significantly decreased in AECs in response to hyperoxia (Figure 3C&D). These results lead to the hypothesis that another posttranslational mechanism of degradation different from shedding is responsible for the decrease in ACE-2 in AECs in response to hyperoxia.

One of the important mechanisms for eukaryotic proteins degradation is through the ubiquitin-proteasome system (UPS). In UPS pathway, proteins are first ‘tagged’ by multimers of a protein known as ubiquitin and are later proteolyzed by a giant enzyme known as the proteasome.^18^ Ubiquitin is a 76-amino acid regulatory protein that can be attached to other proteins at either a lysine residue or to the N-terminus by the consecutive actions of E1, E2, and E3 enzymes. Ubiquitin can also be attached to itself, resulting in poly-ubiquitin chains.

Ubiquitination affects substrate proteins in different ways, for example by resulting in degradation of the substrate protein by the 26S proteasome. Many proteins involved in either the ubiquitination, deubiquitination or degradation of proteins are implicated in human diseases and are currently under investigation as potential drug targets.^19^ In this study, we present evidence of ACE-2 ubiquitination, as shown in figure 4A, with ubiquitinated ACE-2 immunoprecipitate bands appearing at three molecular weights. One band around 100kDa corresponding to ACE-2 expected MW, and two others at 50 and 250 kDa. In literature, there are reports of lower molecular weight ACE-2 degradation product that could explain the 50 kDa band.^20,21^ A potential explanation of the 250 kDa band could be that ACE-2 is poly-ubiquitinated. In addition, we show that hyperoxia increases total protein ubiquitination in AECs (Figure 4B).

Consistent with our results, other studies have found a role of ubiquitination and small ubiquitin-related modifier-1(SUMO1) in hyperoxia exposure models of BPD. In a neonatal rat pups model of BPD, Jing et al. found reduced GTP cyclohydrolase 1 (GCH1) levels in hyperoxia. This reduced GCH1 was due to increased degradation by the ubiquitin-proteasome system.^13^ Zhu et al. found that hyperoxia may induce over-proliferation and differentiation disorders of alveolar epithelial cells in preterm rat model of BPD, possibly through an increased expression of SUMO-modified C/EBPα.^14^ In endothelial cells and rat models of pulmonary arterial hypertension (PH), Shen et al. described that maladapted ubiquitination of ACE-2 is thought to be involved in the pathogenesis of PH.^15^ In a study of neurogenic hypertension, elevated Ang-II levels reduced ACE-2 expression and activity in Neuro-2A cells by stimulation of lysosomal degradation where Ang-II treatment enhanced ACE-2 ubiquitination.^16^ These studies together with our results show an important role that ubiquitination, specifically of ACE-2, could be playing in BPD pathogenesis. Future studies will be directed to characterize this role.

**Figure 6:**
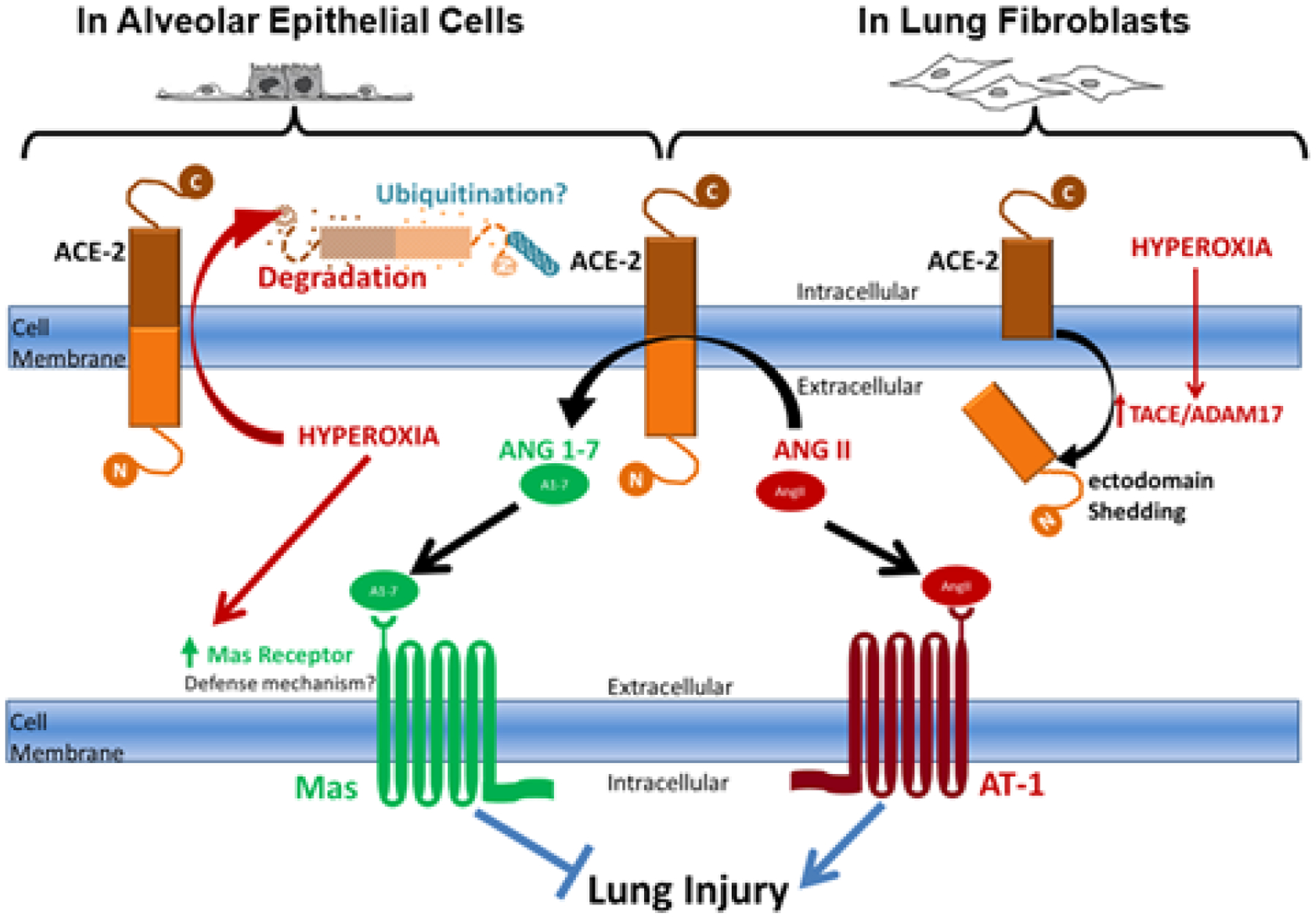
Schematic diagram summarizing the working hypothesis illustrating different pathways for ACE-2 processing and degradation by hyperoxic exposure that are cell-type dependent.

Another interesting result presented here is that hyperoxia increases Mas receptor protein levels (Figure 5). Similar results on the mRNA levels were found by Wagenaar et al. as hyperoxia increased neonatal rat pups Mas mRNA in postnatal day 6.^6^ In that study and our previous study Mas receptor was shown to be protective against hyperoxia-induced lung injury as activation of Mas restored hyperoxia-induced loss of lung epithelial barrier function through inhibition of apoptosis, while agonists of Mas attenuated cardiopulmonary disease in rats with neonatal hyperoxia-induced lung injury.^1,6^ In our working hypothesis (Figure 6) we suggest that a potential explanation could be that Mas is increasing in a negative feed-back mechanism, where hyperoxia is decreasing ACE-2 levels and activity causing a decrease in its Ang1-7 product. This decrease in Ang1-7 signal could be stimulating an increase in its receptor (Mas) to compromise the decreased peptide levels. This supports further detailed investigations of Mas agonism as a potential therapy of BPD.

### Summary and working hypothesis

Our previous studies showed that AngII mediates the profibrotic effect of hyperoxia on cultured human lung fibroblasts and that ACE-2, which protects against lung fibrosis in animal models by degrading AngII, is downregulated in the lungs of patients with Idiopathic Pulmonary Fibrosis through mechanisms yet to be fully identified. Recently, we showed that ACE-2, is decreased in fetal lung fibroblasts in response to hyperoxic gas exposure through a TACE/ADAM17 shedding mechanism. In contrast, we show here that Epithelial cells have different mechanisms to regulate ACE-2. We hypothesize that hyperoxia induces ACE-2 degradation through the ubiquitin proteasome pathway and that the decrease in ACE-2 causes the epithelial cells to increase Mas receptor abundance as a defense feedback mechanism to compensate for Ang1-7 deficiency that would result from ACE-2 degradation.
